# Characterization of temporary and permanent 3D-printed crown and bridge resins

**DOI:** 10.2340/biid.v12.43584

**Published:** 2025-05-02

**Authors:** Roope Salonen, Sufyan Garoushi, Pekka Vallittu, Lippo Lassila

**Affiliations:** a Department of Biomaterials Science and Turku Clinical Biomaterial Center (TCBC), Institute of Dentistry, University of Turku, Turku, Finland; b Wellbeing Services County of South-West Finland, Turku, Finland

**Keywords:** 3D resin, mechanical properties, wear, printing orientation

## Abstract

**Purpose:**

The aim of this study was to evaluate the mechanical, surface, and optical properties of two 3D-printed crown and bridge resins (CROWNTEC and Temp PRINT). Additionally, the study assessed the effects of printing orientation and accelerated hydrothermal aging on their mechanical properties.

**Materials and methods:**

Specimens were 3D-printed using digital light processing technology (Asiga MAX™). Mechanical properties, including flexural strength (FS), compressive strength, and fracture toughness (FT), were determined for each material following ISO standards. Three printing orientations (0°, 45°, and 90°) were used for fabricating 3-point bending specimens. Surface hardness was evaluated using a Vickers indenter. Two-body wear tests were conducted using a ball-on-flat configuration in a chewing simulator with 15,000 cycles, and wear depth was measured with a non-contact 3D optical profilometer. Disk-shaped specimens (*n* = 5/material) were prepared to measure translucency parameter, gloss and light penetration. For gloss measurement, specimens underwent laboratory-machine polishing (4,000-grit abrasive paper) and chairside two-step hand polishing (Top Dent DiaComposite). Posterior composite crowns (*n* = 10/material) were fabricated and subjected to cyclic fatigue aging (5,000 cycles at Fmax = 150 N) before quasi-static loading to fracture. The microstructure of each material was analyzed using scanning electron microscopy (SEM). Data were statistically analyzed using ANOVA and Tukey’s HSD test.

**Results:**

Hydrothermal aging, printing orientation, and material type significantly affected the FS values (*p* < 0.05). Temp PRINT showed superior FS (129 MPa) and FT (1.3 MPa m^1/2^) compared to CROWNTEC (102 MPa, 0.9 MPa m^1/2^), particularly at 0° orientation. Gloss measurements revealed no significant differences between materials (*p* > 0.05) across used polishing systems. SEM analysis demonstrated differences in microstructure between the materials.

**Conclusion:**

Temp PRINT demonstrated superior mechanical performance compared to CROWNTEC, which exhibited higher translucency values. The printing orientation was identified as a critical parameter influencing the mechanical properties and overall performance of 3D printed restorations.

## Introduction

Three-dimensional (3D) printing has many possible uses in dentistry, many of which are already utilized to a certain degree [1]. For example, 3D printing techniques can be used in fabricating dental models, surgical guides, occlusal splints and dental restorations [1, 2]. There are numerous advantages offered by 3D printing technology in producing dental restorations. 3D printing is a form of additive manufacturing, meaning that the printed product is created layer by layer, whereas the more traditional CAD/CAM milling is subtractive manufacturing, meaning that the product is created by removing existing material [3]. Thus, compared to CAD/CAM milling, 3D printing reduces manufacturing time, produces less waste and doesn’t require consumable burs [4, 5]. On the other hand, 3D-printed products show lower strength compared to products made by subtractive manufacturing methods [4].

There exist several technologies for additively manufacturing dental devices: material extrusion, material jetting, powder bed fusion and vat photopolymerization. Due to the long printing times and lower resolutions of material extrusion technologies, they are currently considered to be of lesser relevance. Material jetting methods provide a possibility to use multiple colors, and they yield the smoothest surface. Powder bed fusion makes the use of metals possible in dental additive manufacturing. Among vat photopolymerization technologies, stereolithography (SLA) and digital light processing (DLP) currently stand out as most used in dentistry [6, 7]. SLA is a process where an ultraviolet laser uses a scanning movement to polymerize a layer of liquid photopolymer on the build platform. After each layer, the platform moves downwards, and a new layer of liquid resin is introduced on top of the old one. The laser then cures another layer, and continues until the print is finished [8]. There also exists printers where the platform moves upwards from the bottom of the resin vat. This ‘bottom-up’ movement has been proven superior to the ‘top-down’ movement in many ways, and most current SLA printers utilize it [8, 9]. DLP printing is, in many ways, similar to SLA printing; but instead of a scanning laser, several mirrors display an image of the whole layer at once, which is then polymerized on the build platform. This lowers the fabrication time compared to SLA printing [9, 10].

Lately, various commercial light-curing 3D printing resins for provisional and permanent restorations have been introduced to the market. These materials should exhibit necessary mechanical and esthetic properties to achieve a successful restoration. Some important mechanical qualities are high flexural strength, hardness, toughness and low wear rate [11]. Optical qualities like gloss and translucency are necessary to achieve a natural looking restoration. Manufacturers of 3D printed resins offer a wide range of materials, with some designed for temporary applications and others for permanent restorations. Although both types may be used for restorative purposes, each product might exhibit distinct mechanical, physical, and esthetic properties. This variability raises an important question: How can we determine the optimal 3D-printed resin for specific clinical needs, given the differences in their composition and performance?

Temp PRINT (GC Corporation, Tokyo, Japan) is a light-curing, dimethacrylate-based 3D resin designed for fabricating temporary crowns and bridges. According to the manufacturer, it incorporates advanced filler and anti-sedimentation technologies, making it ideal for precise and long-term provisional use [12]. On the other hand, CROWNTEC (Saremco Dental AG, Rebstein, Switzerland) is a light-curing, methacrylic acid ester-based 3D resin developed for fabricating permanent restorations, including crowns, inlays, onlays, and veneers. It is also suitable for temporary bridges. According to the manu-facturer, the resin offers high esthetics and ease of use [13].

To the author’s knowledge, CROWNTEC is currently one of the few 3D printing resins approved for definitive dental restorations. Other existing permanent resins, like VarseoSmile Crown plus (BEGO, Bremen, Germany) have been studied more extensively [4, 14, 15]. While only a limited number of studies have investigated its mechanical and optical properties, the relative novelty of CROWNTEC and the variability in reported results warrant further comprehensive testing [16, 17]. Previous research has also presented inconsistent findings regarding the impact of printing orientation on the strength of 3D-printed restorations [18, 19].

Therefore, the aim of this study was to characterize the permanent material and evaluate its mechanical properties (flexural strength, compressive strength, fracture toughness, and fracture load), hardness, wear and optical properties (translucency, light transmission, and gloss), while comparing the results to those of the temporary material. Additionally, the study aimed to further explore the effect of printing orientation on the strength of 3D-printed materials.

The null hypotheses were: (1) there is no significant difference in the tested properties between the materials, and (2) printing orientation does not significantly affect the flexural strength of the material.

## Materials and methods

### Specimen preparation

A DLP printer (Asiga MAX^TM^, SCHEU-DENTAL GmbH, Iserlohn, Germany) was used to fabricate the specimens from the two resins. Shade A1 was used for Saremco CROWNTEC (SC), and for GC Temp PRINT (TP), color ‘light’ was used. The specimens were arranged on the build platform and sliced using Asiga Composer 2.0.3 software. Up to date versions of material-specific INI-files were used as printing parameters. Necessary printing supports were automatically generated by the software. The printing files were transferred to the printer to start the printing process. The layer thickness for both materials was 50 μm, as per manufacturers’ instructions. After printing, the specimens were removed from the build platform, washed and post-cured.

Both materials used different washing procedures to get rid of excess resin on the surface of specimens. For CROWNTEC, the specimens were submerged in a container filled with mixed cleaning concentrate (Saremco Dental AG). The solution was prepared by mixing one part of concentrate with four parts of deionized water, as per manufacturer’s instructions. The container was then placed in an ultrasonic water bath (Quantrex 90, L&R Manufacturing, USA) for 3 minutes. After 3 minutes, the specimens were removed, rinsed with warm water and dried. They were then placed in an unused container of mixed cleaning concentrate, and placed in the ultrasonic bath for another 3 minutes. Subsequently, the specimens were again removed, rinsed with warm water and dried. The dry specimens were then post-cured in a light-curing oven (Otoflash^®^ G171, Bego GmbH&Co, Bremen, Germany) under nitrogen gas for 2 × 2,000 flashes, flipping the specimens over after the first 2,000 flashes. The amount of flashes and use of nitrogen were recommended by the manufacturer.

For Temp PRINT, the washing procedure used isopropanol (IPA) instead of the cleaning concentrate. The printed specimens were submerged in a container filled with IPA. The container was then placed in an ultrasonic water bath for 2 minutes. The specimens were subsequently removed, dried and placed in a fresh container of IPA, and placed in the ultrasonic water bath for another 2 minutes. After the second bath, the specimens were removed and dried. The dry specimens were post-cured in an Otoflash light-curing oven under nitrogen gas for 2 × 400 flashes, flipping the specimens over after the first 400 flashes. The amount of flashes and use of nitrogen were recommended by the manufacturer.

In addition to the 3D-printed specimens, experimental groups (*n* = 10/group) of bar-shaped specimens were fabricated by directly light-curing the resins. The resin was injected to a special 3-piece mold. Starting from bottom, the setup included a baseplate, mylar sheet, the mold, another mylar sheet and a microscope glass. The microscope glass was pressed hard to prevent air bubbles from forming in the resin. A curing light (D-Light Pro, GC Corporation, Tokyo, Japan) was placed on top of the microscope glass and used to cure the resin for 3 × 20 seconds on both sides. The specimens were then removed and post-cured in a light-curing oven as per manufacturers’ recommendations.

### Flexural strength measurement

**Figure 1:**
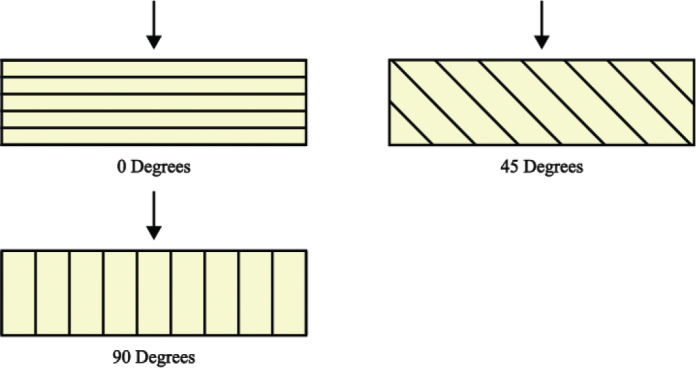
Explanation of the different printing orientations of flexural strength specimens.

For evaluating the flexural strength (FS) following the ISO 4049 guidelines [20], a 3-point bending test was conducted on bar-shaped specimens (*n* = 10/group) measuring 2 × 2 × 25 mm. For both materials, tested printing orientations were: 0 degrees, 45 degrees and 90 degrees (Figure 1). Also, the directly light-cured specimens were tested. There were a total of two groups tested per each orientation for each material. One group was dry-stored (48 hours) at room temperature before testing. The other group was subjected to accelerated hydrothermal aging by placing the specimens in boiling water for 16 hours before testing [21]. Prior to testing, the dimensions of each specimen were measured using a digital caliper. The 3-point bending test was performed with a universal testing machine (LLOYD LR30KPlus, Lloyd Instruments^TM^Ametek^®^ Inc, Largo, Florida, USA) under ambient conditions at room temperature. The load cell capacity was 2,500 N, preload was 1 N and the preload speed was 10 mm/min. Distance between the supports under the specimen was 20 mm. Each specimen was tested until failure, and PC software (Nexygen 4.0, Lloyd Instruments Ltd) was used to record the load-deflection curve. Flexural strength was calculated using the formula: σ = 3FL/(2bd^2^), where F is the load at maximum load (N), L is the length of the support span (mm), b is the width (mm) of the specimen, and d is the thickness of the specimen (mm). Additionally, Young’s modulus was calculated using the formula: *E* = FL^3^/4bh^3^δ, where δ is the deflection at maximum load (mm).

### Fracture toughness measurement

**Figure 2:**
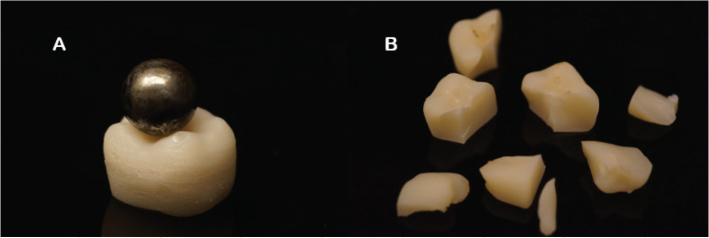
A notched specimen for fracture toughness measurement (left) and the test setup (right).

To evaluate the fracture toughness, bar-shaped specimens (*n* = 6/group) measuring 2.5 × 5 × 25 mm were tested. Single-edge notch specimens were prepared by attaching printed specimens to a custom micromotor rig. A sawblade (spun at 15,000 rpm) was then used to cut a 2.5 mm deep pre-crack in the specimen. The handpiece moved on rails to ensure cutting precision, and the sawblade was water-cooled to reduce heat and warping. After cutting, the notch was measured using a microscope (Wild M3Z, Wild Heerbrugg, Switzerland) and calibrated microscope camera software (ToupCam, Touptek Photonics Co., Ltd.) to ensure correct length of the notch. Finally, a razor blade was passed in the notch 10 times to create an ‘infinitely sharp’ tip for it (Figure 2). The specimens were then either dry-stored or hydrothermally-aged before testing. The hydrothermal aging procedure was the same as with FS specimens. Tested printing orientations were 0 and 45 degrees. The orientations were decided based on flexural strength results.

The specimens were tested with a 3-point bending method, using a universal testing machine with a load cell of 2,500 N, preload of 1 N and preload speed of 10 mm/min. The specimens were numbered and their notch-side was colored with a marker to aid in further measurements. The specimens were tested until failure, and subsequently, the length of the propagated crack was measured from the middle and both edges of the crack. The fracture toughness was calculated using the Equation: Kmax = [P L/B W3/2] *f*(*x*), where: *f*(*x*) = 3/2 × 1/2 [1.99-*x* (1-*x*) (2.15–3.93*x* + 2.7 × 2)]/2 (1 + 2*x*) (1-*x*)^3/2^ and 0 < *x* < 1 with *x* = a/W. Here P is the maximum load in kilonewton (kN), L is the span length (2 cm), B is the specimen thickness in centimeters (cm), W is the specimen width (depth) in cm, x is a geometrical function dependent on a/W and a is the crack length in cm.

### Compressive strength measurement

To evaluate the compressive strength (CS), cylinder-shaped specimens (*n* = 6/group) with dimensions of 4 × 4 × 6 mm were tested. For each material, a dry-stored group and a hydrothermally-aged group were tested. A 0 degree printing orientation was decided based on flexural strength results. The compression test was performed with universal testing machine under ambient conditions at room temperature. The load cell capacity was 30 kN, preload was 4 N and preload speed 5 mm/min. Each specimen was tested until failure. CS was calculated using the formula CS = 4F/πd^2^, where F is the maximum applied load (N) and d is the diameter of the specimen (mm).

### Fracture load test

**Figure 3:**
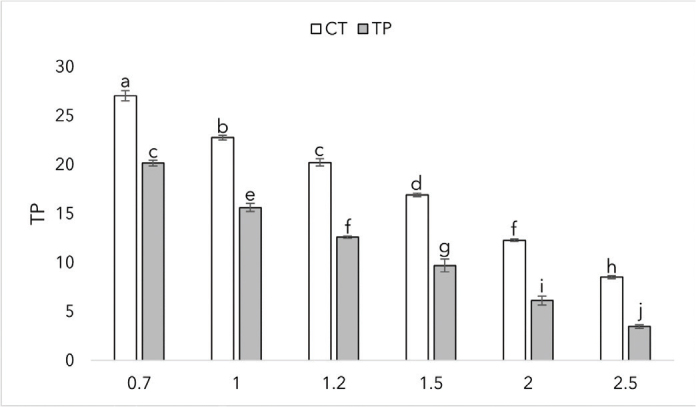
Solid crown with metal ball prior to testing (A) and fragments of fractured crown after testing (B).

Fracture load testing was performed on solid, lower first mandibular-shaped crowns (*n* = 10/group), meaning that the core and the crown were printed seamlessly from the same material (Figure 3). The specimens underwent cyclic fatiguing for 50,000 cycles with a maximum force of 150 N using CeraTest 2k machine (SD Mechatronik, Feldkirchen-Westerham, Germany). During cycling, the specimens were immersed in water. After cycling, quasi-static load was applied to the specimens using a universal testing machine with a speed of 1 mm/min. The loading was vertically applied between the triangular ridges of the buccal and lingual cusps, using a metal ball with a diameter of 5 mm. The specimens were loaded until failure.

### Vicker’s hardness measurement

The Vicker’s hardness value (VHN) was measured from the same specimens used in testing flexural strength. Specimens were tested in 0 degree orientation, based on flexural strength results. The specimens (*n* = 5/group) were polished with a 4,000 grit silicon carbide paper using a water-cooled automatic polishing machine (Struers Labopol-21, Struers A/S, Denmark). The specimens were then superglued to a metal platform. The platform was attached to a surface materials tester (Rtec SMT-5000, Rtec Instruments INC). A suitable indentation area on the specimen was found using the tester’s microscope. A Vicker’s tip was then used to create 3 indents per specimen under 10 N load using a dwell time of 30 seconds. The diagonals of the indentation were measured using the tester’s software. The equation HV≈0.1891*(F/d^2^), where F is the force applied (N) and d is the average length of the diagonals (mm), was used to calculate VHN.

### Translucency parameter measurement

The translucency parameter (TP) was evaluated using disc-shaped specimens (*n* = 5/group) with a diameter of 8 mm and thicknesses of 0.7 mm, 1.0 mm, 1.2 mm, 1.5 mm, 2.0 mm and 2.5 mm. The specimens were polished with a 4,000 grit FEPA silicon carbide paper using a water-cooled automatic polishing machine. The CIELAB color scale was used in evaluating the specimens’ color. The measurements were conducted using a spectrophotometer (CM-700d, Konica Minolta Sensing Inc., Japan) over black and white backgrounds. The spectrophotometer was calibrated for the backgrounds before testing. From the measured L, A and B values, TP-values were calculated using the equation TP = ((L_B_ – L_W_)^2^ + (A_B_ – A_W_)^2^ + (B_B_ – B_W_)^2^)^1/2^, where TP is the translucency parameter, B refers to the color coordinates over the black background and W refers to the color coordinates over the white background.

### Light transmission measurement

To measure light transmission, the same discs that were used in testing translucency parameter were used. The specimens were placed on the bottom surface of a Marc^®^ Resin Calibrator (Blue Light Analytics Inc.). A light-curing device (D-Light Pro) was held directly on the specimen. A stand and a clamp were used to hold the curing light in place. The light was turned on for 20 seconds. The resin calibrator software measured the irradiance in real-time. After the test, the software measured the mean irradiance for different thicknesses.

### Gloss test

Disc-shaped specimens (*n* = 5/group) with a diameter of 10 mm and thickness of 3 mm were used to evaluate surface gloss at 60° using a glossometer (Zehntner ZGM 1110, Zehntner Testing Instruments, Switzerland). Different surface finishes were tested: unpolished, polished to 4,000 grit and a clinical 2-step polishing kit (Top Dent DiaComposite). For 4,000 grit, a silicon carbide paper was used with a water-cooled automatic polishing machine. The 2-step polishing kit came with a medium and a fine polishing tip. The polishing was carried out using a dental unit (Planmeca Advanced Simulation Workspace, Planmeca Oy, Helsinki, Finland) under water cooling for 40 seconds each with a speed of 6,000 rpm.

### Wear test

A two-body wear test was performed on the same disc-shaped specimens (*n* = 4/group) as the gloss test. The specimens were fixed to an acrylic resin block and polished with silicon carbide paper with grain sizes up to 4,000 grit FEPA. The specimens were then stored in water at 37 degrees Celsius for 24 h before testing. A two-chambered chewing simulator (CS-4.2, SD Mechatronik, Feldkirchen-Westerham, Germany) was used to conduct the wear test in the presence of water. The specimens were attached to the lower plastic holder of the simulator, while the manufacturer’s standard loading tips (Steatite ball, 6 mm) were secured to the upper one with a fastening screw. A chewing simulation was performed at 1.5 Hz with a vertical weight of 2 kg, which is equivalent to 20 N of chewing force. The specimens were subjected to 15,000 loading cycles. The steatite ball was changed after each experiment. A 3D optical microscope (Bruker Nano GmbH, Berlin, Germany) was used to scan the wear patterns. The material loss estimates were calculated using Vision64 Map software (version 1, Bruker Nano GmbH, Berlin, Germany). The total vertical depth values were acquired in micrometers (μm) from several sites by averaging the deepest points of all profile scans.

### Microstructure analysis

Scanning electron microscopy (SEM, LEO, Oberkochen, Germany) was used to characterize the microstructure of the investigated 3D resins (magnifications: 2,500× and 5,000×). Polished disc specimens (*n* = 2) from each material (0-degree, post-processed, non-aged) were stored in a desiccator for 1 day. The specimens were then coated with a gold layer using a sputter coater in a vacuum evaporator (BAL-TEC SCD 050 Sputter Coater) before SEM examination. The operating voltage was set to 20 kV, with a working distance of 12.6 mm.

### Statistical analysis

The data were analyzed using JMP Pro 17 (JMP Statistical Discovery LLC, Cary, NC, USA) using ANOVA with significance level of *p* = 0.05. Tukey’s HSD post hoc test was used to find the differences between groups. Normality was analyzed visually and with QQ-plot.

## Results

**Figure 4:**
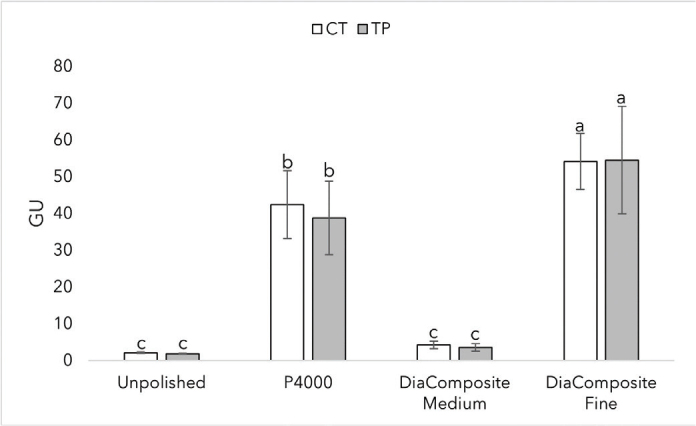
Flexural strength with SD of CROWNTEC (CT) and Temp PRINT (TP) specimens tested under different printing orientations. Different letters indicate statistical significance.

The mean values of the results of the flexural strength test (FS) with standard deviations (SD) are summarized in Figure 4. There were statistically significant differences between the materials, printing orientations and aging. Temp PRINT (TP), printed at 45 degrees, demonstrated the highest FS rating (117 MPa). However, between the dry TP groups, this result was only statistically significant compared to the 90 degree group (*p* < 0.05). CROWNTEC (CT) demonstrated its highest FS rating (102 MPa) in the 0 degree group. This result was also only statistically significant compared to the 90 degree group (*p* < 0.05) when comparing the dry CT groups. In each printed orientation, the dry FS results were higher than the hydrothermally aged FS results. The difference was statistically significant (*p* < 0.05). There was no statistically significant difference in FS between dry and hydrothermally aged groups in the directly light-cured specimens.

With both CT and TP, the hydrothermally aged directly light-cured groups demonstrated higher FS values than hydrothermally aged 3D-printed groups. The difference was statistically significant in each printing orientation.

**Figure 5:**
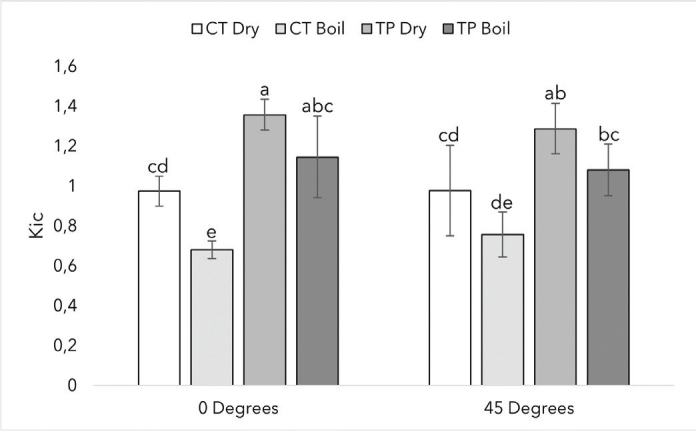
Fracture toughness with SD of CROWNTEC (CT) and Temp PRINT (TP) specimens tested under different printing orientations and artificial aging. Different letters indicate statistical significance.

Figure 5 shows the fracture toughness values of the two materials, with different printing orientations and artificial aging. The highest fracture toughness values for both materials were achieved dry with no statistical significance between printing orientations (*p* > 0.05).

The compressive strength results are represented in Table 1. TP reached higher compressive strength than CT when comparing both dry and aged specimens. The difference was statistically significant (*p* < 0.05). In both materials, strengths between dry and aged groups didn’t differ statistically (*p* > 0.05).

**Table 1:** Compressive strength, hardness, wear and fracture load with SD of the tested materials.

Value	CROWNTEC	Temp PRINT
Dry compressive strength (MPa) mean ± SD	194.33 ± 26.70^b^	288.82 ± 20.43^a^
Boil compressive strength (MPa) mean ± SD	162.58 ± 14.49^b^	312.35 ± 36.23^a^
Vicker’s hardness (VHN10) mean ± SD	24.29 ± 0.94^b^	22.63 ± 1.72^a^
Wear depth (μm) mean ± SD	44.4 ± 3.3^b^	19.3 ± 3.6^a^
Fatigued solid crown fracture load (N) mean ± SD	1605.38 ± 143.27^b^	3194.41 ± 549.19^a^

Different letters indicate statistical significance.

Vicker’s hardness values, wear depth and fracture load of the two different materials are found in Table 1. There was a statistically significant difference in hardness between materials (*p* < 0.05), with CT having higher surface hardness than TP. There was a statistically significant difference in wear depth between the materials (*p* < 0.05), with CT having deeper wear depth than TP. In addition, TP had a higher fracture load with a load of 3,200 N against CT with a load of 1,600 N. The difference was statistically significant (*p* < 0.0001).

**Figure 6:**
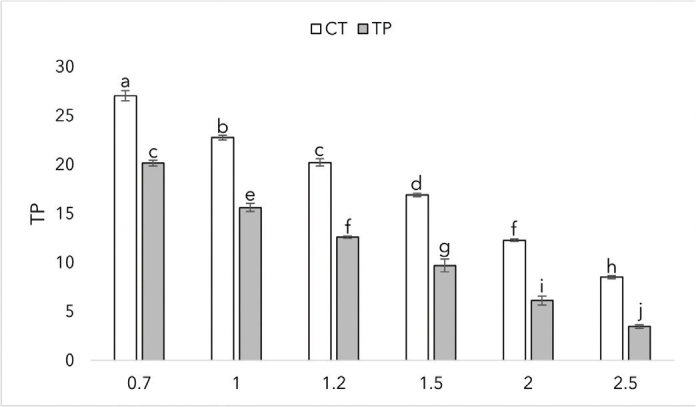
Translucency parameters of CROWNTEC (CT) and Temp PRINT (TP) at different thicknesses (in mm) with SD. Different letters indicate statistical significance.

The translucency parameters of the two materials at different thicknesses are summarized in Figure 6. With both materials, the translucency parameter decreases with increasing thickness. Both materials indicated minimal deviation at given thicknesses. CT reached the highest translucency parameter value of 27 at the thickness of 0.7 mm. At each given given thickness, there was a statistically significant difference between the materials (*p* < 0.0001).

**Figure 7:**
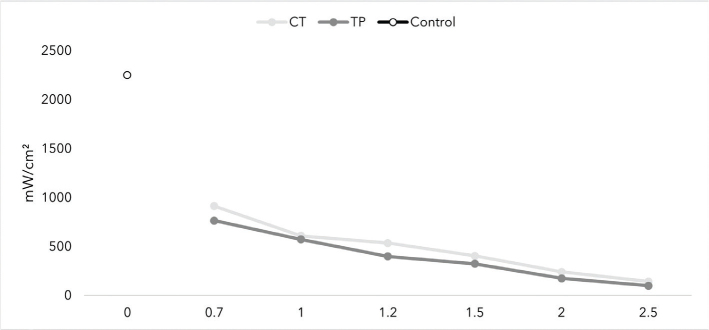
The mean irradiance of CROWNTEC (CT) and Temp PRINT (TP) at different thicknesses (in mm).

Figure 7 represents the irradiance values of the two materials, showing that mean irradiance decreases with increasing thickness, as with the translucency parameter. Thus, both materials showed highest irradiance values at the thickness of 0.7 mm.

**Figure 8:**
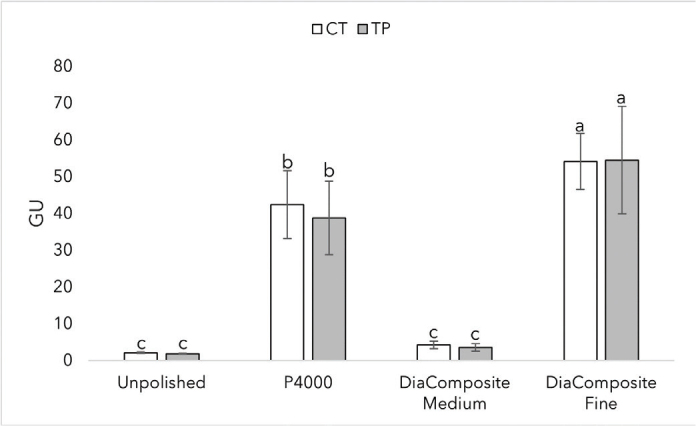
Gloss values of CROWNTEC (CT) and Temp PRINT (TP) with SD at different finishing protocols. Different letters indicate statistical significance.

Figure 8 summarizes the gloss values of CT and TP with SD at different finishing techniques. Highest values were reached using the fine tip, 54 GU for both materials with no statistically significant difference between materials (*p* > 0.05). Using different finishing protocols, all finishes differed statistically (*p* < 0.0001); however, within each specific protocol, the materials did not differ significantly.

**Figure 9:**
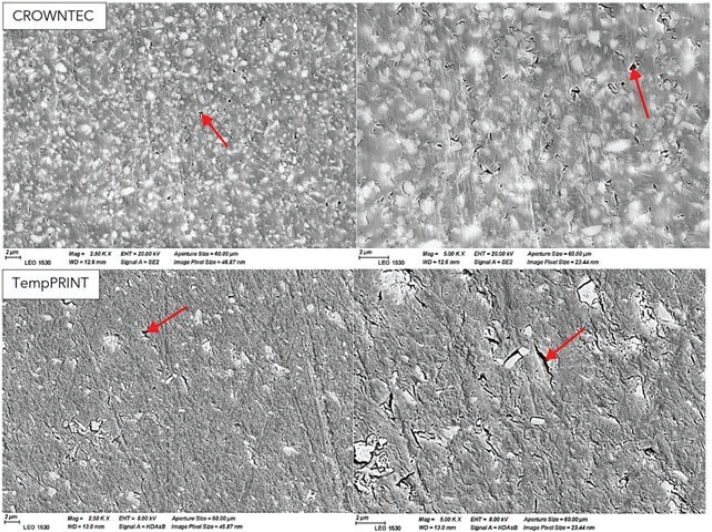
SEM-images of 0-degree, post-processed, non-aged CROWNTEC and Temp PRINT specimens. Arrows indicate voids and small pits.

SEM images of the composition of the two materials are presented in Figure 9, where the inorganic filler particles are visible within the resin matrix. The images indicate that CT has a higher inorganic filler content than TP, which aligns with the stated composition of the materials in Table 2. Additionally, SEM analysis revealed a substantial number of voids and small pits in both materials.

**Table 2:** Materials used in this study with their manufacturer details.

Material	Manufacturer	Lot	Composition	Inorganic filler content
CROWNTEC	Saremco Dental AG	F428	Esterification products of 4,4’-isopropylidiphenol, ethoxylated and 2-methylprop-2enoic acid, silanized dental glass, pyrogenic silica, initiators	30–50% by mass
Temp PRINT	GC Corporation	2405241	Urethane dimethacrylate, silicon dioxide, initiator, pigment, stabilizer	10–25% by mass

## Discussion

According to the results, both of the null hypotheses can be partially rejected. CROWNTEC (CT) and Temp PRINT (TP) exhibited statistically significant differences between their mechanical and optical properties. However, there were situations where the materials didn’t differ statistically, hence the partial rejection of the first null hypothesis. The second null hypothesis can be partially rejected, as printing orientation influenced mechanical properties in some instances, while in others, no significant effect was observed.

As 3D-printed dental restorations face different stresses in the oral cavity, it is of great importance to be able to evaluate a material’s mechanical performance under these stresses. Thus, flexural strength is one of the most important mechanical properties of a dental biomaterial. CT and TP showed differences in their flexural strengths, with TP exhibiting higher values (117 MPa) compared to CT (102 MPa). This can be attributed to the differences between the materials’ composition. As shown in Table 2, the composition of TP is based on urethane dimethacrylate, or UDMA. On the other hand, CT is based on Bis-EMA. It has been shown that dental polymers containing more UDMA are stronger than polymers containing more Bis-EMA [22, 23], and this study follows the same line. Higher filler content has also been attributed to higher flexural strength [18]. However, in this study CT, having higher filler content, has conversely lower flexural strength. This can likely be due to the aforementioned difference in the polymer matrix between the materials. It has to be noted, however, that there was moderate variance in the results, and a statistical difference between materials was only found in two groups. The results were somewhat in line with prior research, and the differences were likely due to differing methodology, such as post-curing, and the inherent characteristics and variation of the materials [16, 24, 25].

Hydrothermal aging is an experimental procedure used to simulate aging of a material. While hydrothermal aging doesn’t take into account, for example, forces of mastication in a material’s aging, and the process in this study was accelerated (boiling), using prolonged higher temperatures than natural, it still gives us insight into how a material could behave in prolonged intraoral use. In this study, hydrothermal aging approximately halved the flexural strength in both materials. Some degradation and separation was also visible on the aged specimens. Due to observed matrix separation, we hypothesized that the inherent layered structure of the printing process might be responsible. To test this hypothesis, specimens from both materials were prepared using a conventional three-point bending mold and a light-curing device. These specimens exhibited a seamless structure, as the entire thickness was cured simultaneously rather than layer-by-layer. In the directly light-cured groups, no statistically significant difference in flexural strength (FS) was found between the dry and boiled conditions. However, the FS of boiled, directly light-cured specimens was significantly higher than that of the printed specimens. These findings suggest that the layered structure of 3D-printed specimens may compromise FS under hydrothermal aging conditions. Literature supports these results, as water uptake has been shown to reduce the mechanical properties of resin materials [2]. Furthermore, 3D-printed resin materials may be particularly vulnerable to water-induced changes, since water can infiltrate between printed layers and alter the interfacial bonding [26].

Fracture toughness is the ability of a material to resist propagating fracture from a pre-fracture when loaded. As dental restorations may chip and develop micro-fractures over time, fracture toughness is a very important property when evaluating a material’s suitability for prolonged intraoral use. In both tested printing orientations, TP had higher fracture toughness values (K_Ic_) than CT. This is in line with prior research, showing that UDMA-based polymers have higher K_Ic_ values than Bis-EMA-based [27]. Boiling reduced the K_Ic_ values, however, a statistically significant difference was observed only in the CT 0-degree group. This outcome may be attributed to variability within the data and the relatively small sample size of the test. Restorations face compressive forces in the oral cavity, so it is important to simulate these forces in research. In this study, we conducted a compressive strength test on printed cylinders and printed fatigued solid crowns. In the cylinder-shaped test, TP was stronger than CT. However, there was no statistically significant difference between the dry and boiled groups in either of the materials, conversely to the FS test. This can be due to the fact that the cylinder-shaped specimens were printed so that the layers were perpendicular to the load. Thus, water absorption-induced separation of the layers didn’t affect the load-bearing capacity of the specimens, as the layers were pressed together.

Fracture load testing was conducted on solid crowns, where the core and crown were printed as a seamless unit from the same material. While this does not fully replicate clinical applications, it provides valuable insight into the material’s mechanical performance under load. Additionally, the specimens underwent artificial fatigue to simulate intraoral conditions. The results indicate that, although not directly comparable, the fracture loads observed were similar to or exceeded those reported for other commercially available 3D-printed resins [28]. Notably, TP exhibited a higher fracture load than CT.

Surface hardness is the ability of a material to resist indentation or penetration [29]. Harder materials resist damage to their surface better, preventing deformation and abrasive damage, thus increasing their longevity as a restorative material. Both materials showed similar hardness values. When comparing with earlier studies, these materials showed similar results to other 3D-printed resins [30, 31]. However, the values are considerably lower than other restorative dental materials, like zirconia or ceramics [32, 33]. It should be noted that Demirsoy et al., achieved notably higher surface hardness values for CT in their study [34], though the results are not completely comparable due to the differences in force and dwell time.

For prolonged intraoral use, evaluating a material’s wear resistance is crucial. Excessive wear can lead to the loss of a restoration’s intended shape and function, while the resulting debris may pose biocompatibility concerns. In this study, CT exhibited approximately twice the wear depth of TP. However, direct comparisons with existing literature are challenging due to methodological differences [35, 36]. Previous studies have shown that filler content influences wear depth in dental composites, with higher filler content generally associated with increased wear [37, 38]. Tamura et al. [37]. reported that wear depth increases alongside improvements in mechanical properties. However, our findings did not align with this trend, as CT exhibited higher wear and lower flexural strength than TP, whereas TP demonstrated the opposite behavior. Our wear results align with the findings of Mangoush et al., who reported that TP exhibited the lowest wear depth among the tested composites [39]. They attributed this to the resiliency of the composite matrix, which provides some shock-absorbing ability. However, composite wear is a complicated process and so far no clear consensus could be found in the literature [40]. Results in literature vary: some studies found a positive [41, 42] or a negative [21, 43] correlation between surface hardness and wear resistance, others did not find an interdependency [44, 45]. Some other studies found that wear resistance of the composite can be influenced not only by the fillers’ size and volume or inter-particle distance, but also by the composition of the resin matrix and by the silane coupling agent which improve the bond of fillers and resin matrix [45, 46]. Furthermore, a number of researchers have hypothesized that fracture toughness and elastic modulus, could also be indicators of clinical wear [47].

When considering a 3D resin for restorative treatment, the optical properties of the material play an important role in achieving a successful and satisfactory outcome. For example, the translucency of a 3D-printed crown has to be similar to human enamel to achieve a natural look. The results show that Bis-EMA based CT has higher translucency than the UDMA-based TP at a given thickness. While we can’t evaluate the effect of filler content on translucency in this research, the results show us that different polymers have different translucency values, and our results are in line with prior research when comparing Bis-EMA and UDMA [48]. At the manufacturer stated thickness of 1.5 mm, CT showed similar translucency values to human enamel [49]. Adequate light transmission is an important property of a restorative dental material. The manufactured crowns can be cemented using light-curing cement, so the material has to let enough light go through to achieve a successful cementation. Both CT and TP showed irradiance to be inversely proportional to thickness. At 1.5 mm, CT and TP reached intensity values of 405 mW/cm^2^ and 324 mW/cm^2^, respectively. The results meet the ISO standard of 300 mW/cm^2^ for resin-based composites [50]. 3D-printed restorations should meet the gloss values of human teeth to achieve a glossy, natural look. At a given surface finish, the gloss values (GU) of the two materials didn’t statistically differ. Values of 40–60 GU is typical level of desired gloss [51]. CT achieved the desired gloss on 4,000 grit, and both CT and TP achieved the desired gloss on the finishing tip of the DiaComposite 2-step kit. The results show that both of the materials have to be polished to achieve an esthetically pleasing outcome.

3D-printing opens the possibility to manufacture dental appliances at different printing orientations. As the printed layers can be in different orientations compared to the force exerted on the restoration, it is fair to suggest that different printing orientations can affect the mechanical properties of a 3D printed material. For CT, the flexural strength differed statistically when comparing the 0-degree group and the 90-degree group, with the 90-degree group having lower values. For TP, flexural strength also differed statistically when comparing the 0-degree group and the 90-degree group, with the 90-degree group having lower values. These results are in line with previous studies, showing that printing orientations closer to 0 degrees, so the layers are perpendicular to the load, exhibit higher flexural strength [52–54]. One possible explanation could be the fact that the layered composition of a specimen could provide a route for a crack to propagate between the layers, this being most evident in the 90-degree printing orientation, where the layers are parallel to the load. Rather conversely to the flexural strength results, we found no statistical difference between printing orientations in fracture toughness. However, this is in line with a previous study [55]. To the authors’ knowledge, the affect of printing orientation on fracture toughness of 3D resins remains largely unexplored.

As 3D-printers print in vertical direction, printing orientation greatly affects printing duration. 90-degree orientations take usually the longest time to print, as they are tallest. Additionally, we noticed that 90-degree specimens can be more prone to moving during printing, especially if the printing supports are inadequate. This is most likely due to their smaller surface area for attachment compared to 0- or 45-degree orientations. We also noticed that the 45-degree printing orientation required most material for the printing supports. Given these insights and the test results, 0-degree printing orientation should be considered as the primary printing orientation, due to its strength and saves in printing time and material usage.

In SEM analysis, a substantial number of voids and small pits were observed in both materials, which can be attributed to the layer-by-layer nature of the 3D-printing process, resin viscosity, and filler distribution. The presence of voids may result from incomplete polymerization between layers, or residual solvents that evaporate over time. These findings highlight potential limitations in the structural integrity of 3D-printed dental composites and suggest that optimizing printing parameters and post-processing protocols may help reduce void occurrence.

Due to differences in methodology, comparison with existing literature isn’t straightforward. For example, Sahin et al., and Temizci et al., reached much higher FS values of 217 MPa and 233 MPa respectively [56, 57]. However, Sahin et al., used different span length (12 mm) and Temizci et al., used a different method altogether (biaxial flexural strength). Studies with comparable methodology achieved typically a little higher FS for CT, for example Alhotan et al., and Di Fiore et al. [58, 59]. Mangoush et al., achieved similar FS for TP [60]. Mudhaffer et al., achieved similar FS with CT, but noticeably lower FS with TP [24]. To the authors’ knowledge, fracture toughness hasn’t been evaluated for CT before. TP behaved similarly to Mangoush et al. [60]. When comparing microhardness values, we found differences in force and dwell time between studies [56, 59, 61]. Temizci et al., used similar force but shorter dwell time, and achieved similar results for CT [57]. Sasany et al., achieved similar translucency values for CT in their studies [62, 63], and Mangoush et al., for TP [64]. As 3D-printed restorations are still a relatively new subject, literature would benefit from more uniform methodology to aid in comparing results between studies.

The results of this study give us insight on the suitability of these materials for clinical use from mechanical and optical perspectives. The results show that CT and TP perform similarly to other 3D resins on the market. TP performed mechanically better than CT, and can be considered a better choice for restorations that require higher strength, for example posterior restorations or temporary bridges. As it is designed to be temporary, its worse optical performance is of less importance. Also, better mechanical performance is generally favored over esthetics in posterior restorations. CT could be considered a more esthetic resin, due to its higher translucency. Higher translucency is especially favored in anterior restorations to achieve a natural appearance. Less translucent materials will be more noticeable on the dental arch, as they will look too opaque and less detailed. Too high translucency will also appear as unnatural. CT, imitating translucency of human teeth better than TP, can thus be considered a better choice for more esthetic restorations. Also, anterior restorations generally require less strength than posterior restorations. It can be said then that TP is a feasible choice for posterior, high-strength restorations, while CT would be a better choice for anterior, esthetics-driven restorations. However, its lower mechanical performance raises the question of how is it marketed as a permanent resin while TP is marketed as a temporary resin. Also, CT had lower flexural strength than the manufacturer-stated 130 MPa [13]. On the other hand, TP had higher flexural strength than the manufacturer-stated 90 MPa [12].

Lastly, the limitations of this study must be considered. Firstly, only two materials were evaluated, without comparisons to other permanent materials against CT or other temporary materials against TP. This limits the generalizability of this study. Accelerated hydrothermal aging doesn’t fully replicate intraoral aging, which limits the usability of the results from a clinical viewpoint. As specimen preparation was carried out with multiple batches across many days, it could have been susceptible to human error, for example regarding the preparation of the resin or post-processing. Additionally, the sample sizes may be a limiting factor. While mechanical and optical properties are important, they represent only a portion of a restorative material’s overall clinical performance. Other critical factors, such as cytotoxicity and solubility, were not assessed in this study, limiting the clinical relevance of this study. Considering the limitations of this study, future research should focus on comparing more 3D-printed resins, additional critical properties and long-term clinical performance of these materials.

## Conclusions

Within the limitations of this study, we conclude that Temp PRINT performed better mechanically, while CROWNTEC had more favorable optical properties. 0-degree printing orientation should be primarily considered for dental 3D-printing. The layered structure resulting from the printing process compromised the flexural strength of 3D-printed specimens when subjected to hydrothermal aging.
